# Supplementary material for: Modelling Skylarks (Alauda arvensis) to Predict Impacts of Changes in Land Management and Policy: Development and Testing of an Agent-Based Model
Source: PLoS One. 2013 Jun 6;8(6):e65803. doi: 10.1371/journal.pone.0065803 (PMC3675089; doi:10.1371/journal.pone.0065803)
Supplement: Supporting Information S4 — The skylark ODdox as a zipped archive. (ZIP) [file pone.0065803.s004.zip › Skylark_ODdox/struct_int_array100.html]

ALMaSS Skylark ODdox: IntArray100 Struct Reference


ALMaSS Skylark ODdox
 2.0


- Main Page
- Related Pages
- Classes
- Files

- Class List
- Class Index
- Class Hierarchy
- Class Members

Public Attributes

IntArray100 Struct Reference

A struct of 100 ints.
More...

`#include <populationmanager.h>`

List of all members.

|  |  |
| --- | --- |
| Public Attributes | |
| int | n [100] |

---

## Detailed Description

A struct of 100 ints.

---

## Member Data Documentation

int IntArray100::n[100]

---

The documentation for this struct was generated from the following file:

- populationmanager.h


- IntArray100
- Generated on Thu Jan 10 2013 13:15:36 for ALMaSS Skylark ODdox by
   1.8.1.1
